# Coriander Genomics Database: a genomic, transcriptomic, and metabolic database for coriander

**DOI:** 10.1038/s41438-020-0261-0

**Published:** 2020-04-01

**Authors:** Xiaoming Song, Fulei Nie, Wei Chen, Xiao Ma, Ke Gong, Qihang Yang, Jinpeng Wang, Nan Li, Pengchuan Sun, Qiaoying Pei, Tong Yu, Jingjing Hu, Xinyu Li, Tong Wu, Shuyan Feng, Xiu-Qing Li, Xiyin Wang

**Affiliations:** 1 Center for Genomics and Biocomputing/College of Life Sciences, North China University of Science and Technology, Tangshan, Hebei 063210 China; 2 School of Genomics and Bio-Big-Data, Chengdu University of Traditional Chinese Medicine, Chengdu, 610075 China; 3 Library, North China University of Science and Technology, Tangshan, Hebei 063210 China; 4 Fredericton Research and Development Centre, Agriculture and Agri-Food Canada, Fredericton, New Brunswick E3B 4Z7 Canada

**Keywords:** Comparative genomics, Genome, Plant evolution

## Abstract

Coriander (*Coriandrum sativum* L.), also known as cilantro, is a globally important vegetable and spice crop. Its genome and that of carrot are models for studying the evolution of the Apiaceae family. Here, we developed the Coriander Genomics Database (CGDB, http://cgdb.bio2db.com/) to collect, store, and integrate the genomic, transcriptomic, metabolic, functional annotation, and repeat sequence data of coriander and carrot to serve as a central online platform for Apiaceae and other related plants. Using these data sets in the CGDB, we intriguingly found that seven transcription factor (TF) families showed significantly greater numbers of members in the coriander genome than in the carrot genome. The highest ratio of the numbers of MADS TFs between coriander and carrot reached 3.15, followed by those for tubby protein (TUB) and heat shock factors. As a demonstration of CGDB applications, we identified 17 TUB family genes and conducted systematic comparative and evolutionary analyses. RNA-seq data deposited in the CGDB also suggest dose compensation effects of gene expression in coriander. CGDB allows bulk downloading, significance searches, genome browser analyses, and BLAST searches for comparisons between coriander and other plants regarding genomics, gene families, gene collinearity, gene expression, and the metabolome. A detailed user manual and contact information are also available to provide support to the scientific research community and address scientific questions. CGDB will be continuously updated, and new data will be integrated for comparative and functional genomic analysis in Apiaceae and other related plants.

## Introduction

*Coriandrum sativum* L. (2*n* = 2× = 22) is an Apiaceae species whose young plants before flowering are usually referred to as cilantro or Chinese parsley, whereas its flowering plants and seeds are generally referred to as coriander. The Apiaceae family exhibits more than 3700 species in 434 genera (https://en.wikipedia.org/wiki/Apiaceae), including several well-known crops such as carrot (*Daucus carota*) and celery (*Apium graveolens*). Coriander is a globally important vegetable crop. Its production tripled from 1994 to 2016, and Asia accounts for 71.4% of its total production worldwide (http://faostat3.fao.org/).

Coriander exhibits high nutrient levels, and is rich in vitamin C and carotene^1^. Coriander leaves and stems are edible vegetables, and its seeds can be used as a spice. In addition, coriander contains several medicinal ingredients and presents important medicinal value. Interestingly, Coriander contains many volatile oils that are responsible for its special aroma, which arouses an unpleasant reaction in 4–14% of people (https://en.wikipedia.org/wiki/Coriander). Scientists have found that most people who dislike coriander share a common olfactory receptor gene, *OR6A2*, whose product absorbs the odor of aldehyde chemicals^2^.

With the rapid development of sequencing technologies, an increasing number of genomes are being sequenced and released^3,4^. Therefore, many plant genome databases have been constructed to allow researchers to search and download omics data sets. Although these statistics are incomplete, there are more than 40 available genome databases for single plants or families such as Arabidopsis (http://www.arabidopsis.org/index.jsp), rice (http://rice.plantbiology.msu.edu), maize (https://www.maizegdb.org)^5^, cotton^6^, carrot^7^, Chinese cabbage^8^, pineapple^9^, and strawberry^10^ (Table 1). In comparison with other families, few genome sequences have been released for the Apiaceae family, and only the carrot genome has been sequenced thus far^11^. However, there is no such database available for coriander, which limits the ability of researchers to obtain genomic and other types of omics data for coriander. Although a carrot genome database has been built, the genome size, gene number, and number of repeat sequences are significantly greater in coriander than in carrot. Therefore, it is necessary to build a genome data analysis platform for coriander together with carrot genome data.

**Table 1:** Summary of main genome databases for genome-sequenced plants

Species	Database	Links
Arabidopsis	TAIR	http://www.arabidopsis.org/index.jsp
Rice	RGAP	http://rice.plantbiology.msu.edu
Maize	MaizeGDB	https://www.maizegdb.org
Soybean	SoyBase	https://soybase.org
Apple	AppleGFDB	http://gfdb.sdau.edu.cn
Cacao	CGD	https://www.cacaogenomedb.org/main
Pineapple	PGD	http://pineapple.angiosperms.org/pineapple/html/index.html
Cabbage	Bolbase	http://ocri-genomics.org/bolbase/
Carrot	CarrotDB	http://apiaceae.njau.edu.cn/carrotdb
Peanut	PeanutBase	https://www.peanutbase.org
Wheat	ATGSP	http://aegilops.wheat.ucdavis.edu/ATGSP/
Pepper	PGP	http://peppergenome.snu.ac.kr
Quinoa	QGDB	http://quinoa.kazusa.or.jp
Chrysanthemum	CGDB	http://www.amwayabrc.com
Chickpea	CGAP	http://www.nipgr.ac.in/CGAP/home.php
Citrus	CAP	http://citrus.hzau.edu.cn/orange/
Cucurbit	CuGenDB	http://cucurbitgenomics.org
Carnation	Carnation DB	http://carnation.kazusa.or.jp
Eucalyptus	ECDB	http://www.kazusa.or.jp/eucaly/
Strawberry	GARDEN	http://strawberry-garden.kazusa.or.jp
Sunflower	SGDB	https://sunflowergenome.org
Sweetpotato	GARDEN	http://sweetpotato-garden.kazusa.or.jp
Morning glory	INGP	http://viewer.shigen.info/asagao/
Cotton	CottonGen	https://www.cottongen.org
Hop	HopBase	http://hopbase.cgrb.oregonstate.edu
Jatropha	JGDB	https://www.kazusa.or.jp/jatropha/
Lettuce	LGR	http://lgr.genomecenter.ucdavis.edu
Medicago	MGD	http://www.medicagogenome.org
Mint	MGR	http://langelabtools.wsu.edu/mgr/organism/Mentha/longifolia
Banana	BGH	https://banana-genome-hub.southgreen.fr
Zoysia	ZGD	http://zoysia.kazusa.or.jp
Panax	PNGD	http://www.plantkingdomgdb.com/panax_notoginseng/
Oak	OGS	http://www.oakgenome.fr
Radish	RGD	http://radish.kazusa.or.jp
Eggplant	EGDB	http://eggplant.kazusa.or.jp
Pennycress	PG	http://pennycress.umn.edu
Vangularis	VIGGS	https://viggs.dna.affrc.go.jp
Brassica	BRAD	http://brassicadb.org/brad/
Rosaceae	GDR	https://www.rosaceae.org
Gramene	Gramene	http://www.gramene.org
Solanaceae	SGN	https://solgenomics.net

Here, we report the construction of the Coriander Genomics Database (CGDB), which contains a high-quality genome assembly of coriander obtained with the most recent sequencing technologies and novel bioinformatics methods. Moreover, this database contains RNA-seq and metabolic data sets from four tissues and three periods for coriander and carrot. The CGDB also provides a large amount of comparative genomic analysis data, such as information on collinear blocks, transcription factors, repeat sequences, and gene annotations. All users can readily access the data using the browser and query a variety of data types from the CGDB, including genomic, annotation, TF, RNA-seq, and metabolic data sets. We hope that CGDB will become an important platform for the plant research community to conduct comparative and functional genomic analyses of coriander and other related plants.

## Materials and methods

### Collection of genome, expression, and metabolic data sets

The genomic, expression, and metabolic data sets included in our CGDB database were obtained from standard experiments and bioinformatics analyses. The genomic data sets mainly contained genome sequence, gene sequence, protein sequence, gff, gene annotation, and repeat sequence data. The expression data sets mainly contained gene expression data for coriander and carrot from three different growth stages and four tissues. The metabolic data sets included information on metabolism genes from coriander and carrot from three different growth stages. All the analysis results for these data sets are easily navigable and available in the CGDB.

### Transcription factor family identification

Transcription factors (TFs) play very important roles in regulating the expression of genes related to plant growth and development and various abiotic stresses. Here, we used the Pfam_Scan.pl program implemented at the Pfam (http://pfam.sanger.ac.uk) database (e-value < 1e-4) to predict TFs in coriander and carrot whole genomes. Then, we conducted the TF classification according to the Pfam results by using Perl scripts.

### TUB gene family identification

The grape (Genoscope 12×) and carrot (v2.0) genome data used in this study were downloaded from the Phytozome website (https://phytozome.jgi.doe.gov/pz/portal.html)^11,12^. Coriander (v1.0) genomics data were derived from our CGDB database. The Pfam database (http://pfam.sanger.ac.uk) was used to perform a domain search on the amino acid sequences, and the genes containing a “TUB” domain (PF01167) were extracted by using a Perl program. ClustalW was used to perform multiple alignments of the sequences of the tubby (TUB) gene family (https://www.genome.jp/tools-bin/clustalw). PhyML 3.0 was employed to construct ML trees with the Jones, Taylor, and Thorton (JTT) model and 1000 nonparametric bootstrap replicates^13^. The pheatmap package of R was used to draw expression clusters (https://cran.r-project.org/web/packages/pheatmap/index.html).

### Genome collinearity detection

To clarify the evolutionary relationships and whole-genome duplication (WGD) or triplication (WGT) events between coriander and five other representative plant species (carrot, lettuce, tomato, grape, and Arabidopsis), we performed a whole-genome comparative analyses. First, the whole-genome protein sequences from all the species were searched against their own sequences using BLASTP with an E-value cutoff of 1 × 10^−5^. Then, MCScanX software (-k 50, -s 5, -m 25) was used to detect the duplicate types and collinearity blocks based on a previous report^14^. Finally, the collinear regions between any two of these plant species were visualized using the Circos and TBtools software packages.

### Database architecture and implementation

**Fig. 1:**
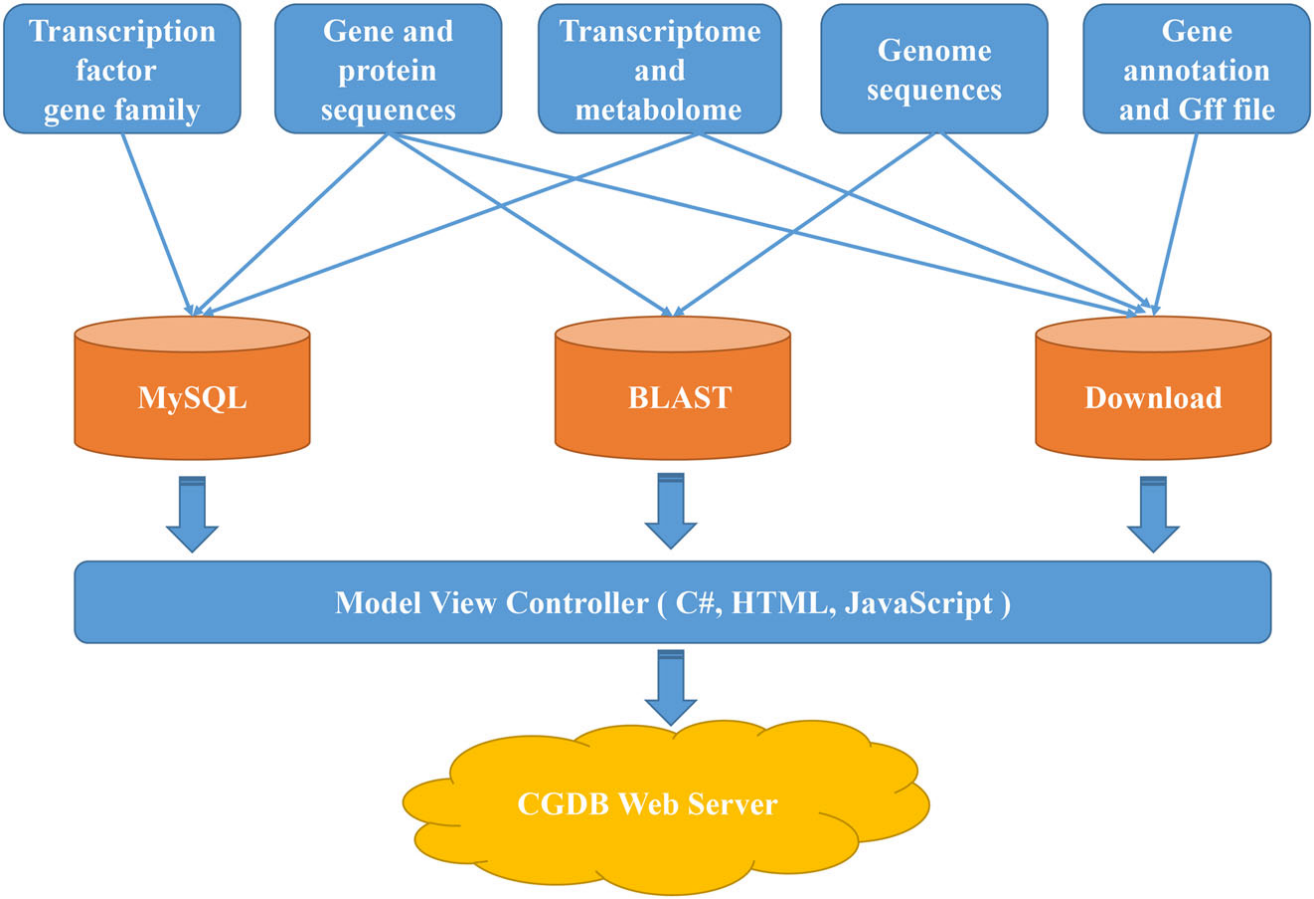
Flowchart of the Coriander Genomics Database (CGDB) sitemap

The CGBD was implemented by applying a variety of common software packages in a Windows system, including internet information services (IIS), MySQL database management, ASP.NET, and HTML. The data were processed and analyzed by using C#, HTML, and JavaScript, and bioinformatics tools were employed for interpreting biological significance. The CGBD consists of relational databases storing processed data in MySQL. An interactive Web interface was constructed to enable users to conveniently access the CGBD and obtain information needed for either basic research applications or biological analysis through any modern browser on their devices. C#, HTML, and JavaScript were implemented to transmit user query information and rapidly extracted data from the MySQL database management system to generate report pages (Fig. 1). The genome browser (JBrowse) was constructed to visualize the genomic data and gene structure^15^. For the interactive alignment of genome sequences, BLAST searches were performed by using BLAST-2.7.1 + , an independent web server for flexible queries of similar nucleotide and amino acid sequences.

## Database construction

### Main interface

**Fig. 2:**
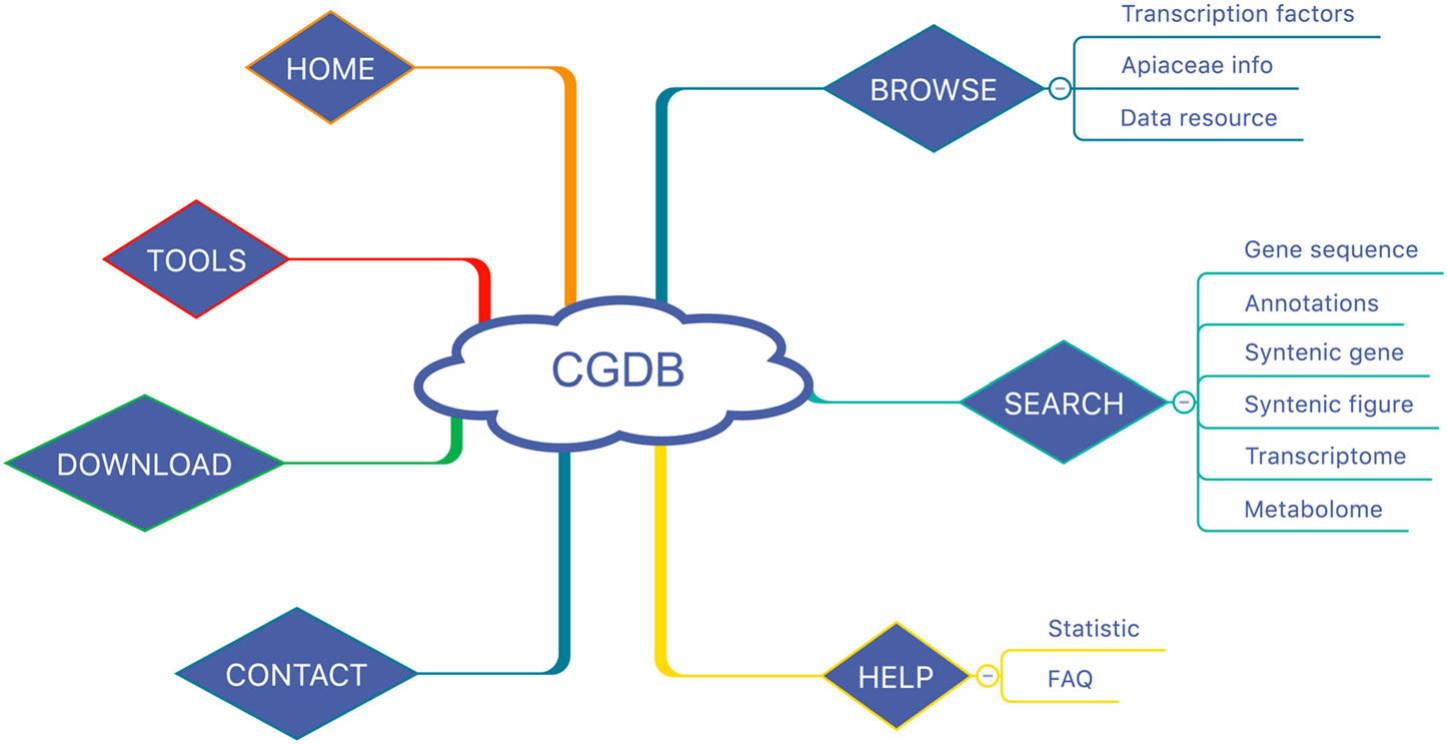
The architecture of the Coriander Genomics Database (CGDB) mainly includes the home, browse, search, tools, download, help, and contact modules

**Fig. 3:**
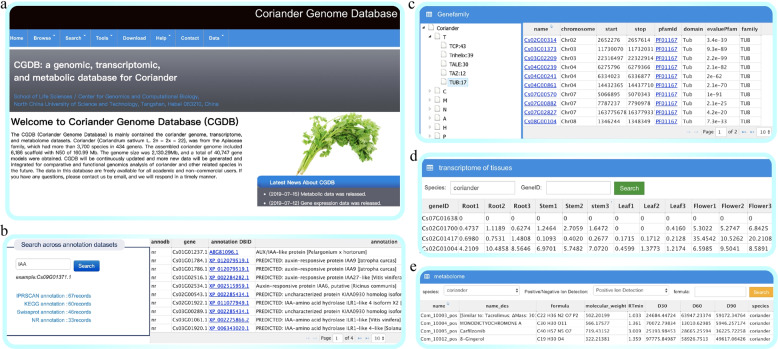
The main functions of the browse and search module. **a** Home page. **b** Gene annotation page. **c** Gene family browser page. **d** Transcriptome data search page. **e** Metabolome data search page

The CGDB structure consists of seven main modules: Home, Browse, Search, Tools, Download, Contact, and Help (Figs. 2 and 3a). In the browse module, we provide the transcription factor data for coriander and carrot, information on the main Apiaceae germplasm resources, and links to the major public genome data resources (Fig. 3c). In the search module, the CGDB allows the users to search the gene sequences, protein sequences, and annotations of the coriander genome by gene names (Fig. 3b). More importantly, users can search the novel transcriptome and metabolome data of coriander and carrot in our database (Fig. 3d, e). Users can obtain gene FPKM values from different tissues and development periods by inputting a gene name from coriander or carrot. Furthermore, the users can search the molecular weights and contents of metabolites determined in coriander plants after 30, 60, and 90 days of growth and in carrot by using the metabolite formula.

**Fig. 4:**
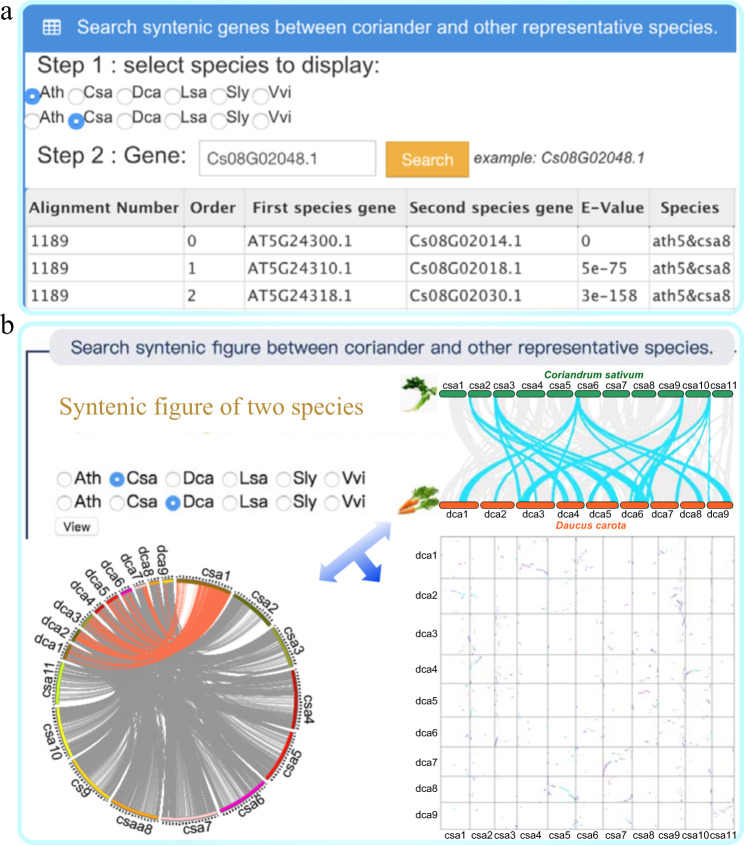
Gene synteny search function of CGDB. **a** A search for syntenic genes. **b** A search for figures illustrating synteny

Furthermore, we provide the syntenic genes and figures illustrating synteny among coriander and five other representative plants (Fig. 4). The online access tool provides the BLAST tool to users, which can search similar sequences on the basis of nucleotide or amino acid sequences. Furthermore, we built a genome browser to interactively display coriander genomic data. In addition, the help module provides data statistics and FAQs to help users to quickly and conveniently use this database. In summary, the CGDB will benefit both comparative genomic and functional genomic studies in plants, especially for coriander and other Apiaceae species.

### Genome sequences and annotation resources

Recently, we sequenced and assembled the coriander genome using PacBio, Illumina, 10X Genomics, and HiC sequencing technology. The assembled genome was 2118.31 Mb, accounting for 99.44% of the estimated genome. The contig N50 length was 604.13 Kb, and the scaffold N50 reached 160.99 Mb. Therefore, we obtained a high-quality assembled coriander genome. To facilitate the convenient use of these data by other researchers, we constructed the CGDB database, and all of the genome and related analytical data sets can be easily downloaded and retrieved from the CGDB by all users.

The CGDB contains not only genome sequences but also 40,747 gene and protein sequences for searching and downloading. Among these genes, 37,772 (92.7%) coriander genes annotated at four databases (Swiss-Prot, TrEMBL, KEGG, and InterPro) were also provided on our CGDB website. In addition, this database includes the annotation of specific repeat sequences, which accounted for 70.59% of the coriander genome. A total of 7233 noncoding RNAs, including 339 miRNAs, 780 tRNAs, 5440 rRNAs, and 674 snRNAs, can also retrieved by a researcher studying noncoding RNAs.

### Expression and metabolic data resources

In addition to the genome data, the CGDB provided the expression and metabolic data for functional genomic studies. The expression data sets were obtained from three different stages and four tissues of coriander by using RNA-Seq. The different growth stages were 30, 60, and 90 days after sowing, and the four tissues were the roots, stems, leaves, and flowers of coriander. Each sample was included three biological replications. In total, 39,225 (90.74%) genes were shown to be actively expressed in at least one tissue, whereas 4005 genes exhibited no expression in the four examined tissues of coriander. A total of 35,759 (83.93%) genes were detected in the transcriptome in at least one stage, while 6848 genes presented no expression among the three developmental stages in coriander. In addition, the expression data for three different growth stages of carrots were included in our database for comparative analyses. In total, 28,667 (82.10%) genes were detected in the transcriptome of at least one growth stage, and 6251 genes exhibited no expression among the three developmental stages of carrots.

Metabolomics is a relatively new discipline and an emerging omics technology that emerged after genomics, metabolomics, and transcriptomics, and has become an important component of systems biology research. Our database integrated the metabolic data sets of coriander and carrots from three different growth stages, including 30, 60, and 90 days after sowing. All of these expression and metabolic data sets are easily navigable and available in the CGDB for all users.

### Transcription factor family data

**Fig. 5:**
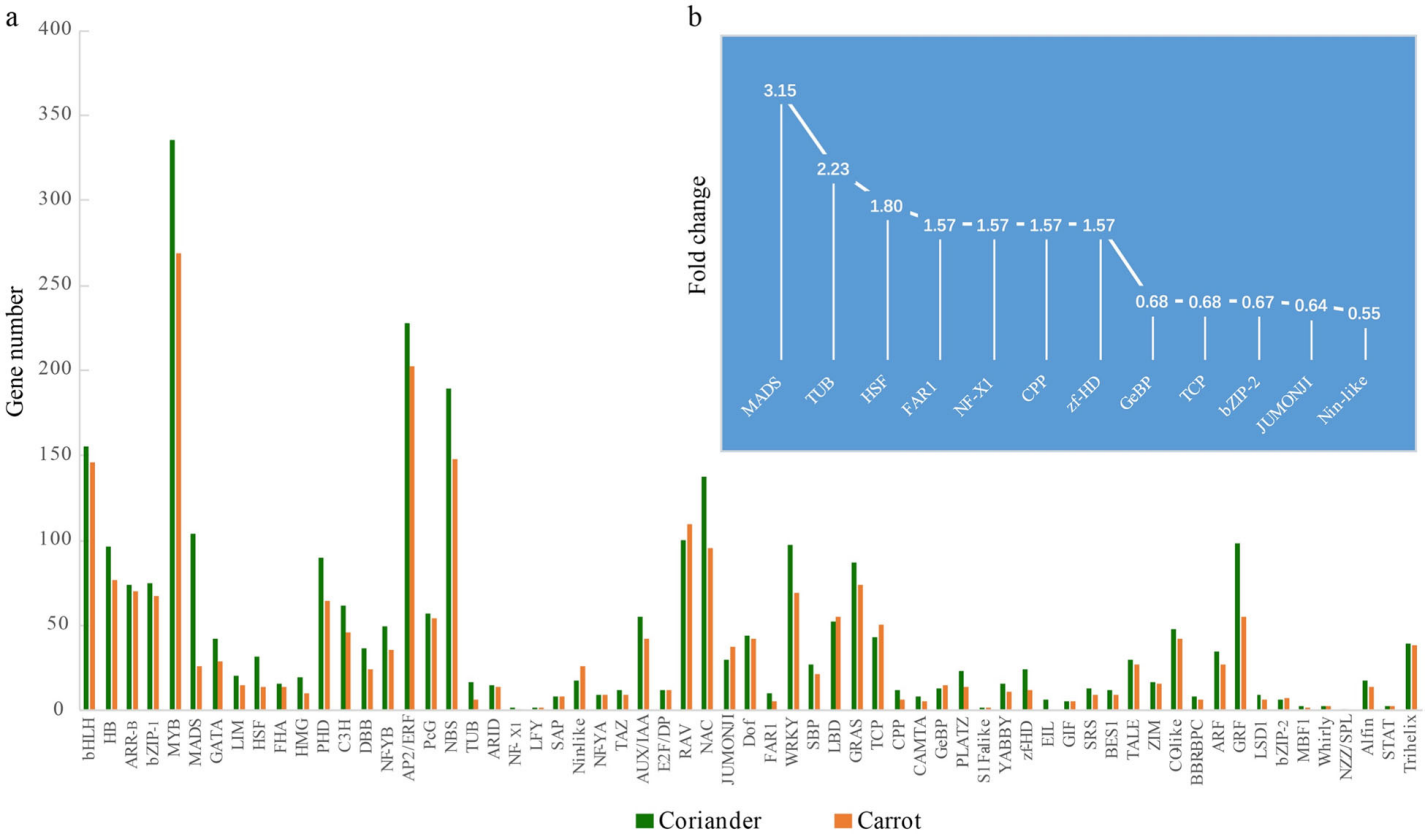
Transcription factor (TF) analyses of coriander and carrot genomes. **a** The number of members of each TF family in these two species. Green represents coriander, and orange represents carrot. **b** The TFs showing the 12 highest ratios of the number in coriander to that in carrot

Transcription factors (TFs) regulate the transcription of downstream genes by binding to their specific DNA sequences. For user convenience, 2908 and 2330 genes were classified into 63 and 61 TF families from the whole-genome sequences of coriander and carrot, respectively, and all of them can be searched in the browse module of the CGDB (Fig. 5a). Two families, EIL and NZZ/SPL, presented six and one genes, respectively, in coriander, while no such genes were detected in the carrot genome. The MYB gene family was the largest among all these predicted TF families, followed by the AP2/ERF and NBS gene families. There were 335/269, 228/202, and 189/148 genes in the coriander/carrot genome for these three large gene families, respectively.

**Fig. 6:**
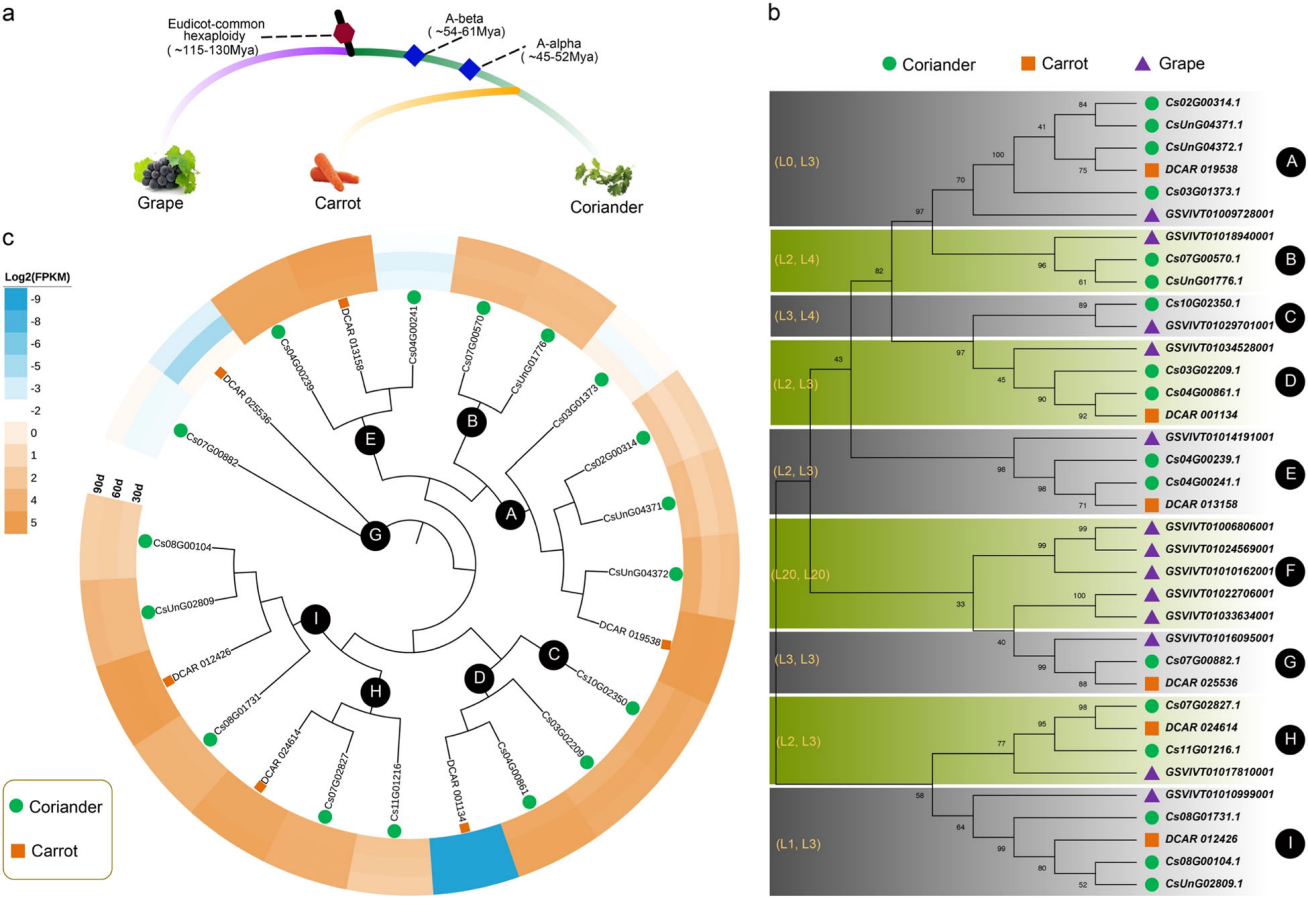
TUB gene family analyses. **a** The evolution and divergence time of grape, carrot, and coriander. **b** Phylogenetic tree constructed using the TUB family genes of these three species. The tree was divided into nine groups, from A to I. The numbers in parentheses represent the number of genes lost in coriander and carrot, respectively. **c** TUB family gene expression analyses of the coriander and carrot genomes at 30, 60, and 90 days after sowing. The FPKM values of gene expression were transformed by log2

Furthermore, we compared the number of TFs for each gene family between the coriander and carrot genomes. The total gene number in coriander (40,747) is ~1.27 times that (32,118) in the carrot genome. Given the difference in the total number of genes in the two species, we first divided the number of TFs in coriander by 1.27 and then calculated the difference in TFs between the two species. The results showed that seven gene families were larger (ratio > 1.5) in coriander than in carrot (Fig. 5b, Table 2). The largest family was the MADS TFs, showing a ratio greater than 3.15, followed by TUB and HSF TFs. These results indicated that these gene families have expanded and might play important roles in coriander development. In addition, five gene families showed lower expression (ratio <0.7) in the coriander genome than in the carrot genome (Fig. 5b, Table 2). All the TFs of coriander and carrot were added to our databases, and TFs from other Apiaceae species will also be provided in the future. All of these DNA/protein sequences and their domain information can be easily searched in the browse module of the CGDB. Therefore, the CGDB provides rich resources for users to conduct gene family analyses. Here, we take the TUB gene family as an example to conduct evolutionary and expression analyses (Fig. 6).

**Table 2:** The number of significantly different transcription factors (TFs) and the ratio of their numbers between coriander and carrot

TFs	Coriander	Corrected coriander^a^	Carrot	Ratio
MADS	104	81.89	26	3.15
TUB	17	13.39	6	2.23
HSF	32	25.2	14	1.8
FAR1	10	7.87	5	1.57
NF-X1	2	1.57	1	1.57
CPP	12	9.45	6	1.57
zf-HD	24	18.9	12	1.57
GeBP	13	10.24	15	0.68
TCP	43	33.86	50	0.68
bZIP-2	6	4.72	7	0.67
JUMONJI	30	23.62	37	0.64
Nin-like	18	14.17	26	0.55

^a^Normalization was conducted due to the different gene numbers found in the whole genomes between coriander and carrot

#### Example of CGDB application: the TUB gene family

A total of 17 TUB transcription factor family members were identified in the whole genome of coriander via the browse module of the CGDB. To investigate the evolutionary relationships of this gene family, we identified 6 and 13 TUB family genes from carrot and grape, respectively (Fig. 6b).

Most genome-wide duplication events, including WGD and WGT events, a accompanied by gene loss or retention^16,17^. To elucidate the evolution of the TUB gene family in coriander, we performed gene loss and replication retention analyses. Comparison with grape showed that two WGD events occurred in coriander and carrot after their divergence from grape^11^. Here, 13 TUB family genes were identified in grape. In theory, there should be 52 TUB family genes in coriander and carrot (13 × 2 × 2), but only 17 and 6 TUB family genes were identified in coriander and carrot, respectively. Thus, these two WGD events did not result in the expansion of the TUB gene family, and significant gene loss occurred in the coriander and carrot genomes.

We detected the quantitative changes in the number of TUB family genes based on the phylogenetic reconstruction obtained for coriander, carrot, and grape. We divided the tree into nine groups (A to F) according to the grape sequence (Fig. 6b). In theory, one grape gene should correspond to four genes of coriander and carrot. However, we found that most groups exhibited gene loss, which ranged from 1 to 4 genes, except in group A of the coriander genome. In carrots, there was more gene loss among all groups, with the number of lost genes ranging from 3 to 4. In particular, there were no genes in the coriander and carrot genomes corresponding to the five grape genes in group F. These results indicated that the loss of TUB family genes occurred in both the coriander and, especially, the carrot genomes.

We also conducted expression analyses of these TUB families using the RNA-seq data deposited at our CGDB website (Fig. 6c, Table 3). Interestingly, we found that the number of TUB family genes was lower in carrot than in coriander in most groups, but their expression was higher in carrots than in coriander, as observed for groups A, E, H, and I. These findings suggest that there may be a dose compensation effect of gene expression in coriander. However, the carrot TUB family gene from group D, *DCAR 001134*, did not show expression in any of the three developmental stages, while two coriander genes from the same group presented a higher expression level than that in coriander.

**Table 3:** TUB gene expression (FPKM) in coriander and carrot at 30, 60, and 90 days after sowing

Species	Gene ID	D30	D60	D90
Coriander	Cs02G00314.1	5.1723	1.8828	2.8621
	Cs03G01373.1	0.5188	0.1765	0.3396
	Cs03G02209.1	18.0678	27.0037	23.3596
	Cs04G00239.1	33.7855	32.1864	31.0476
	Cs04G00241.1	0.1361	0.1078	0.1995
	Cs04G00861.1	27.5520	36.0511	36.4085
	Cs07G00570.1	13.7428	10.7806	13.9516
	Cs07G00882.1	0.1661	0.1518	0.3668
	Cs07G02827.1	19.7302	20.3621	20.7112
	Cs08G00104.1	3.3019	4.1946	3.7648
	Cs08G01731.1	15.4158	16.4117	16.5387
	Cs10G02350.1	20.6233	33.1526	27.9979
	Cs11G01216.1	6.7023	5.2225	4.0803
	CsUnG04371.1	9.9767	3.4971	5.5451
	CsUnG04372.1	21.8226	8.0290	9.6861
	CsUnG01776.1	9.7076	10.1039	9.7378
	CsUnG02809.1	12.3509	12.2642	14.2535
Carrot	DCAR_001134	0.0000	0.0000	0.0000
	DCAR_025536	0.3597	0.0248	0.0985
	DCAR_024614	30.2723	31.2355	36.8822
	DCAR_012426	45.1803	51.8409	47.1426
	DCAR_019538	51.7943	54.2469	54.1548
	DCAR_013158	36.3231	41.8629	46.6245

In conclusion, all of these gene family and expression data sets can be searched and downloaded from the CGDB database. Therefore, similar comparative and functional genomic analyses can be conducted by numerous scientific and agricultural researchers or breeders.

### Integration of comparative genomics data

Coriander, carrot, and celery are Apiaceae species. The carrot genome and a related database have been reported, and its chromosome number (2*n* = 18) is different from that of coriander (2*n* = 22). Therefore, our database provides rich resources for the comparative genomic analysis of Apiaceae plants. From carrot and coriander genome analyses, we found that two WGD events are shared by Apiaceae species (Fig. 6a). The findings from these genomes and the observed duplication events can led us to conduct genome analyses of evolution, polyploidy, and comparative genomics, examining characteristics such as gene collinearity and gene family expansion and contraction.

Functionality for comparative genomic analyses is available in the CGDB database for users who want to conduct homology comparisons and evaluate systematic evolution and duplication events between coriander and other species. The CGDB provides collinear gene pairs and figures illustrating collinearity between any pair of the species *C. sativum*, *A. thaliana*, *D. carota*, *L. sativa*, *S. lycopersicum*, and *V. vinifera*. The collinear regions between the coriander genome and these species are provided by the collinear region search function, and users can query detailed information about collinear genes in the search module of the CGDB website.

### BLAST server and genome browser

The BLAST tool was embedded in the CGDB database using the BLAST-2.7.1+ program to help users perform sequence alignment^18^. We provide a user-friendly graphic interface based on Web forms. The BLAST database contained the whole-genome sequences, gene sequences, and protein sequences of coriander and nine other related species, including carrot, lettuce, Arabidopsis, rice, tomato, potato, grape, kiwifruit, and Amborella. All users can perform similarity searches of genes against each type of sequence by using various BLAST search programs, such as BLASTn, BLASTp, BLASTx, tBLASTn, and tBLASTx. Amino acid or nucleotide acid sequences in FASTA format can be directly submitted by copying the data to the frame or uploading a FASTA file. Some parameters, such as the e-value, score, and output format, can be modified or simply set to the default parameter values by the users according to the research aims before performing a BLAST search. Finally, clicking the search icon provides the link to the results interface for users. The query sequences along with their position in the whole-genome sequences of coriander are produced and ordered according to the expected values, and the user can browse or download the BLAST results.

A genome browser was developed to display the coriander genomic data set and the features and structure of coriander genes. In addition, users can query the genomic sequences of each chromosome, which enables users to enlarge and view relevant information for specific genes, such as gene names, types, positions, lengths, and sequences.

### Data download

The download page was provided to allow users to download entire data sets, including the whole coriander and carrot genomic sequences, gene sequences, protein sequences in FASTA format, and gene structure according to gff results. The gene annotation data set of coriander contains gene functional descriptions from several protein databases, including InterPro, Swiss-Prot, NR, and KEGG, which is provided for users on the download page and search page. Coriander transcriptome data from four tissues (roots, stems, leaves, and flowers) and three different developmental stages of leaves are available to provide expression information for the related genes. In addition, carrot transcriptome data from the three different developmental stages of leaves are available to provide expression information on the related genes. We also provide coriander and carrot metabolome data from the three different developmental stages of leaves.

### Help and contact

In the help module, statistical information and the answers to frequently asked questions (FAQs) are provided for users. The statistical data are provided for three main types of data: genomic data, RNA-seq data, and metabolic data. The genomics data mainly included genome survey data, the data output, Hi-C statistics, chromosome lengths, sequence classifications, and gene annotations. The RNA-seq data included a summary of the data for four tissues in coriander and three different periods in both coriander and carrot. The metabolic data included the formula, molecular weight, and contents of metabolites at the developmental stages of 30, 60, and 90 days after the sowing of coriander and carrot.

In addition, we provide a detailed FAQ manual, which includes the ten main questions that might arise among the users, such as how to download and cite the CGDB. All the main frequent questions and answers can be found in this document. We also provide information such as our contact e-mail, mobile phone numbers, and addresses in the contact module to help users to contact us conveniently and quickly.

### Limitations and future development

Due to the lack of phenotypic trait information for coriander samples, few studies and reports on trait-related markers have been reported. Based on the existing genomics data, a large number of phenotype-related molecular markers, such as SSRs and single-nucleotide polymorphisms (SNPs), will be added to this database in the near future.

Coriander is a dicot and belongs to the Apiaceae plants phylogenetically; the coriander genome is the best genome reported to date for studying the gene family evolution in Apiaceae combined with the carrot genome. The CGDB database will be updated with new data and information in a timely manner. With increasing research on coriander, large quantities of data on transcriptome, metabolome, and proteome sequences and phenomics data will emerge in in the future. We will continuously collect these omics data sets and store them in the CGDB to allow users to conduct further comparative genomic and functional analyses.

Due to the rapid development of software and bioinformatics methods, plus the variety of omics data sets for genes, the structure, and function of the current coriander annotation will be improved in the future. In addition, we will identify molecular markers such as SSRs and SNPs in various genotypes of coriander, which will allow users to conveniently conduct genome-wide association studies (GWAS) and molecular marker-assisted selection (MAS) for coriander. Moreover, we welcome and encourage all users to send us feedback for the further improvement of this database. We believe that the CGDB will be a useful and user-friendly database for coriander researchers and breeders.

## Conclusion

We developed the Coriander Genomics Database (CGDB), which includes a large amount of genomic data and other omics data sets, for research on the coriander genome, transcriptome, and metabolome. The popular, powerful BLAST search tool was implemented, which allows users to search for their target genes in coriander and several other related species in this database. The genome browser can be easily used to search the detailed information of each gene. This site is intended to be the major database for coriander research. The intuitive browser of the CGDB enables searches, visualization, downloading, and the observation of cross-species collinearity, providing the most recent, unrestricted access to various omics data sets among users. We integrated all of these resources into a portal and provided useful coriander and comparative genomic resources. This database will help to establish an active global community of coriander researchers and facilitate comparative genomic evolution studies in Apiaceae.

## Data Availability

The genome assembly and annotation of coriander are available in the CGDB database. The CGDB can be freely accessed at http://cgdb.bio2db.com/ via the internet. All data in this database are freely available for academic research purposes. Newly released data for coriander will be updated at this website. All materials and related data from this study are available upon reasonable request. Enquiries concerning the CGDB database can be directed by email to songxiaoming116@163.com.
